# IL-1 receptor antagonist as a diagnostic biomarker for bacterial infections in acute decompensation of cirrhosis

**DOI:** 10.1038/s41598-025-30647-5

**Published:** 2025-12-07

**Authors:** Georgios Konstantis, Andreas Schütte, Björn Jung, Moritz Passenberg, Lucie Jacquet, Clara Guntlisbergen, Nargiz Nuruzade, Dieter P. Hoyer, Jan Best, Katharina Willuweit, Hartmut H. Schmidt, Sabrina Guckenbiehl, Jassin Rashidi-Alavijeh

**Affiliations:** 1 Department of Gastroenterology, Hepatology and Transplant Medicine, Medical Faculty, University of Duisburg-Essen, Duisburg, Germany; 2 Department of Infectious Diseases, West German Centre of Infectious Diseases, University Medicine Essen, University Duisburg-Essen, Essen, Germany; 3 Department of General, Visceral and Transplantation Surgery, University Hospital Essen, Essen, Germany

**Keywords:** Biomarkers, Diseases, Gastroenterology, Immunology, Medical research

## Abstract

Liver cirrhosis is a chronically progressive disease often leading to severe complications such as acute decompensation (AD) and acute-on-chronic liver failure (ACLF). Both conditions are frequently triggered by bacterial infections. However, conventional biomarkers such as procalcitonin (PCT) and C-reactive protein (CRP) have limited diagnostic accuracy for detecting infections in patients with cirrhosis. The aim of this study was to better characterize the inflammatory response in AD and ACLF and to identify reliable biomarkers for the early detection of bacterial infections. In this prospective study, 74 consecutive patients with liver cirrhosis and either AD or ACLF were enrolled between October 2022 and October 2023 during hospitalization at the University Hospital Essen. Bacterial infection was identified as a precipitating event in 33 patients, whereas other causes were found in the remaining 41 patients. In addition to CRP and PCT, several proinflammatory cytokines and interleukin receptor antagonists were analyzed, including IL-1β, IL-1RA, IL-17E, IL-17F, IL-17A, IL-6 and IL-23, which play central roles in type I and type III immune responses. Univariable logistic regression analyses were conducted to identify biomarkers significantly associated with bacterial infections. Significant biomarkers were then incorporated into multivariable models alongside predefined clinical parameters. Model performance and generalizability were evaluated through internal validation using 1,000-fold bootstrap resampling with 74 patients per sample. Key biomarkers identified included CRP, PCT, IL-1β, and particularly IL-1RA. After dichotomization, the following combinations achieved the highest diagnostic accuracy for bacterial infection: IL-1RA alone (AUC: 0.762; 95% confidence interval [CI]: 0.652–0.872), IL-1RA combined with PCT (AUC: 0.750; 95% CI 0.635–0.866), and IL-1RA combined with CRP (AUC: 0.744; 95% CI 0.625–0.863). To minimize overfitting and improve model calibration, ridge regression was applied. The final model, which included IL-1RA and selected clinical parameters, achieved an AUC of 0.764 (95% CI 0.654–0.874), with an accuracy of 73%, sensitivity of 61%, specificity of 83%, and precision of 74%. Based on this model, a risk stratification tool was developed that defined thresholds to achieve 80% sensitivity and specificity, respectively. A freely available online application was created to facilitate clinical implementation. In summary, the model developed in this study enables improved identification of bacterial infections in patients with liver cirrhosis. This could support earlier and more targeted therapy in this highly vulnerable high-risk population. External validation and application in larger cohorts are necessary to further confirm the clinical utility of the model.

**Supplementary Information:**

The online version contains supplementary material available at 10.1038/s41598-025-30647-5.

## Introduction

In recent years, it has become increasingly clear that advanced chronic liver disease (ACLD) can be categorized into distinct stages, each characterized by specific clinical features and a unique pattern of disease progression^1^. These stages are primarily driven by the progressive rise in portal hypertension, the development of a hyperdynamic circulation, bacterial translocation, and the activation of systemic inflammation^1^. Decompensated cirrhosis represents an advanced stage, marking a critical turning point in a patient’s quality of life, risk of hospitalization, and the overall prognosis^2^. The onset of the symptoms can develop progressively over time or manifest acutely within hours or days^3^. This acute decompensation (AD) can lead to a rapid further deterioration of liver function^4,5^. Acute-on-chronic liver failure (ACLF) is the most severe form of decompensation, recognized as a distinct syndrome^6,7^ and is characterized by complications such as ascites along with the failure of one or multiple organs, including the kidneys, brain, coagulation system, respiratory system, and circulation^8,9^.

Among the various precipitating factors, bacterial infections are the most frequent trigger and are strongly linked to increased mortality^10–13^. One of the key mechanisms underlying the increased susceptibility of patients with ACLD to bacterial infections is cirrhosis-associated immune dysfunction (CAID)^14–16^. CAID is an immune state characterized by chronic systemic inflammation and impaired immunocompetence, rendering patients highly susceptible to infections^14,15,17^. Therefore, gaining a deeper understanding of the defective immune responses associated with CAID is essential for improving risk stratification, optimizing therapeutic strategies, and ultimately enhancing patient outcomes.

On the other hand, current guidelines emphasize the importance of maintaining a high index of clinical suspicion for bacterial infection and recommend procalcitonin (PCT) and C-reactive protein (CRP) as biomarkers of infection^18^. Still, in the context of CAID, these biomarkers can be significantly influenced by systemic inflammation, impaired liver function and sterile inflammation, making their interpretation particularly challenging^19,20^. There is also a significant gap in current guidelines on antibiotic use in patients with ACLF, particularly regarding the role of CRP and PCT in antibiotic stewardship^18,21^. The indiscriminate use of antibiotics or substances that affect the microbiota can also have potentially harmful consequences, leading to an emerging prevalence of multidrug-resistant (MDR) bacterial infections in patients with AD and ACLF^22,23^.

From the above, it becomes clear that biomarkers less affected by CAID and more reflective of immune dysregulation should be developed, as they will provide valuable diagnostic and prognostic insights^18,21^. Therefore, the primary objective of this study was to investigate the relationship between various first- and second-line interleukins (IL), as they hold promise for differentiating bacterial infections from sterile inflammation in patients with cirrhosis. Additionally, we aimed to evaluate their potential as diagnostic biomarkers through a rigorous statistical approach.

## Materials and methods

### Patients

This is a retrospective analysis of a prospective, single‐center cohort study. Between 11.2022 and 11.2023, a total of 74 adult patients with acutely decompensated cirrhosis were consecutively admitted to the University Hospital Essen in Germany. All participants provided written informed consent to be included in the study. The study protocol was approved by the local ethic committee of the University Hospital Essen, Germany, in accordance with the Helsinki Declaration of 1975. In cases where patients were unable to provide consent due to incapacity, informed consent was obtained from a legally authorized representative.

Patients who met the following exclusion criteria were not included: acute liver failure, pregnancy, HIV seropositivity, or evidence of disseminated malignancy. Cirrhosis was defined as the presence of at least two of the following: i) histological confirmation on liver biopsy, ii) laboratory abnormalities consistent with cirrhosis, or iii) radiological signs indicative of cirrhosis and portal hypertension. AD of chronic liver disease and ACLF were classified and graded based on the number of organ failures, following the criteria established in the CANONIC study^8^. All patients were monitored throughout their hospitalization until discharge and subsequently followed for 12 months, or until death or liver transplantation, whichever occurred first.

### Data collection

We gathered data on patient demographics, comorbid conditions and biochemical markers. All patients underwent routine laboratory assessments, including a complete blood count with differential, renal and liver function tests, and microscopic urine analysis. Screening for bacterial infections included a chest X-ray, blood cultures, urine cultures, and ascitic fluid analysis, with additional cultures conducted as clinically indicated, and were diagnosed as per standard criteria^24^. Upon enrollment, all patients were prospectively monitored to ensure that microbiological cultures obtained at the time of inclusion tested negative or positive. Liver disease severity was assessed using the sequential organ failure assessment (SOFA), chronic liver failure consortium (CLIF)-AD, CLIF-ACLF, model of end-stage liver disease (MELD), and Child–Pugh scores. Sepsis syndrome was diagnosed in accordance with the SEPSIS-3 guidelines^25^. Serum was collected at the indicated baseline and follow‐up time points.

### Quantification of cytokines

Serum concentrations of IL-17A, IL-17F, IL-17E, IL-23, IL-1β, IL-1RA, and IL-6 were quantified using the Quantikine enzyme-linked immunosorbent assay (ELISA) kit (R&D Systems, Minneapolis, MN) following the manufacturer’s instructions. Sample and standard curve values were measured at 450 nm using an EnVision plate reader.

### Statistics

Data characteristics were summarized as frequencies and percentages for categorical variables and as medians with interquartile ranges (IQR) for continuous variables, owing to their non-normal distribution. Comparisons between groups were performed using the Wilcoxon rank-sum test for continuous variables and the Pearson Chi-square test for categorical variables. To explore initial cytokine patterns and reduce dimensionality, principal component analysis (PCA) was performed using all cytokine data. Correlations among interleukins were visualized to elucidate their interrelationships and guide the selection of variables for regression models. Univariate logistic regression analyses were first conducted for all possible biomarkers. Results were expressed as Odds Ratios (OR) with 95% Confidence Intervals (CI). Biomarkers that remained significant after adjustment were further evaluated to determine their potential clinical utility.

### Development and evaluation of a biomarker-based score for infection diagnosis

To develop and validate a clinical diagnostic model, we followed a structured methodology^26^. First, we identified the need for a new tool and defined its purpose and intended application (step 1 and step 2). We then assessed the quality and quantity of our dataset (Step 3), ensuring the inclusion of all relevant predictors such as patient history (e.g., ACLF status, MELD score), clinical and laboratory parameters, and cytokine profiles. For model development (step 4), we employed robust statistical techniques. Univariate logistic regression analyses were conducted as aforementioned. The model was designed to generate infection risk predictions ranging from 0 to 100% (step 5). Given the absence of established thresholds for biomarkers such as IL-1β and IL-1 receptor antagonist (IL-1RA), we sought to identify optimal cut-off values to assess their association with infection. Logistic regression was employed with each biomarker dichotomized (≤ cut-off vs. > cut-off) as the sole predictor. Optimal cut-off values were determined by minimizing the Akaike Information Criterion (AIC), following established methodologies^27^.

The model’s performance was then compared against widely used clinical biomarkers such as CRP and PCT, both individually and in combination. To evaluate predictive performance (Step 6), we focused on four key domains: *discrimination, calibration, validation,* and *overall performance.* Discrimination was assessed using the area under the receiver operating characteristic (ROC) Curve (AUC) with 95% confidence intervals. We also conducted two analyses to evaluate calibration—the calibration belt and the calibration plot. Finally, we validated the model using bootstrapping to adjust for apparent optimism in performance. The clinical utility of the model was assessed using a decision curve analysis. All statistical analyses were performed using R software (version 4.2), with a significance level of 0.05 applied for all tests.

## Results

### Clinicopathologic characteristics of patients

**Fig. 1:**
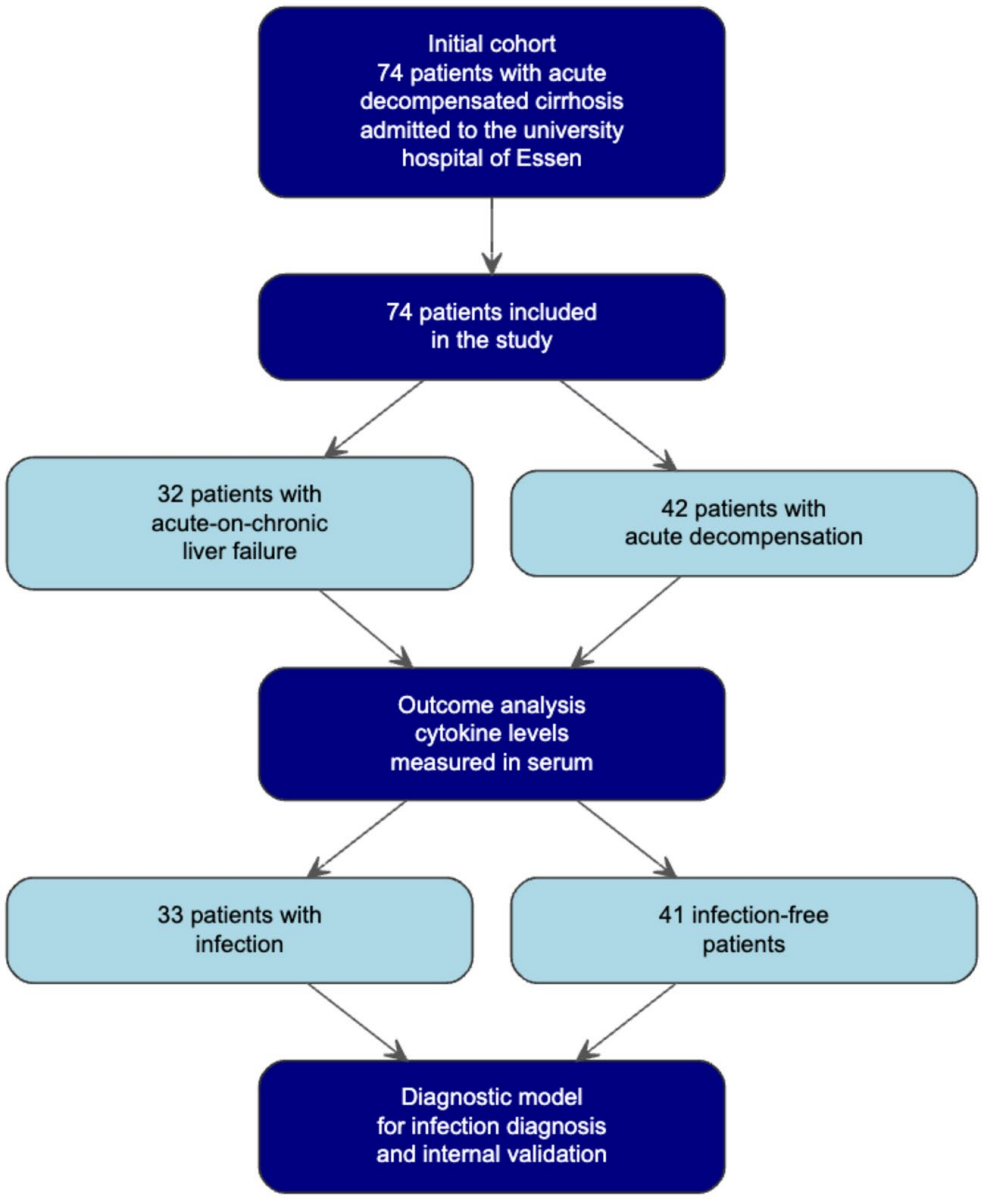
Flow diagram.

We included 74 patients with acute decompensation in the study. Of these, 41 patients had no infection and 33 had confirmed infection, diagnosed on the basis of positive blood or urine cultures, radiological evidence, or immunological tests. The study flowchart is presented in Fig. 1. Patient demographics, clinical characteristics, and laboratory data are summarized in Table 1. Patients with infections were younger than those without, with a mean age of 52 ± 12 years, and 45% were male. Additionally, patients with infections had a lower BMI (25.68 kg/m^2^ ± 6.83) compared to those without infections (29.15 kg/m^2^ ± 5.96) (p = 0.007). Among patients with ACLF, 27 (47%) had ACLF grade 1 (23 of whom had renal failure only), 13 (23%) had ACLF grade 2, and 17 (30%) had ACLF grade 3 at the time of enrolment, while the remaining patients had AD without ACLF. Infections were present at inclusion in 7 (14%) of patients with AD and in 26 (46%) of patients with ACLF. No significant difference was observed between the two groups regarding MELD score (21.94 ± 8.72 vs. 19.22 ± 8.12). However, patients with infections exhibited significantly higher levels of IL-17 pg/mL (331.56 ± 555.89 vs. 119.76 ± 376.12, p = 0.004), IL-1RA pg/mL (1047.14 ± 994.05 vs. 453.80 ± 649.03, p = 0.02), IL-1β pg/mL (60.20 ± 84.70 vs. 16.00 ± 45.40, p = 0.002), and IL-23 pg/mL (1797.25 ± 2788.65 vs. 551.07 ± 1632.37, p = 0.01). Elevated levels of CRP (4.66 mg/dl ± 4.08; 2.69 mg/dl ± 2.62) and PCT (1.08 ng/ml ± 2.04; 0.37 ng/ml ± 0.60) were also observed in patients with infections (p = 0.009 and 0.005 respectively). Similarly, higher levels of bilirubin, alkaline phosphatase, and ferritin were noted in patients with infections. The majority of patients in both groups suffered from alcohol-induced cirrhosis. The exact causes of cirrhosis are shown in Supp. Table 1. The group without infection had statistically significantly more patients with Metabolic dysfunction-associated steatohepatitis (MASH)-induced cirrhosis (9 vs. 2, P = 0.01). The interleukin concentrations between the two groups are presented in Supp. Figure 1. Regarding the site of infection, eight patients (25%) had an unclear bacteremia, which was attributed to bacterial translocation, probably due to severe portal hypertension. Seven patients (21.5%) presented with urinary tract infections, while five patients (16%) were diagnosed with cholangiosepsis. Additionally, seven patients (25%) suffered from pneumonia, two (6.25%) from peritonitis, and two (6.25%) from Clostridioides difficile colitis (Supp. Table 2). In patients diagnosed with pneumonia based on clinical and radiologic findings, microbiological confirmation was not achieved in all cases. Among the identified pathogens, Escherichia coli was the most frequently isolated organism (n = 10; 37%), followed by Enterococcus faecium (n = 5; 19%) and Staphylococcus aureus (n = 3; 12%). Less commonly, Klebsiella oxytoca and Clostridioides difficile were each detected in 2 patients (8%). Other organisms included Pseudomonas aeruginosa, Acinetobacter baumannii, Staphylococcus haemolyticus, and Serratia marcescens (each n = 1; 3.6%) (Supp. Table 2).

**Table 1:** Demographic, clinical and laboratory features of patients at admission according to group.

Variable	Infection (mean ± SD) n = 33	No Infection (mean ± SD) n = 41	P-value		
Age (years)	52.48 ± 11.79	56.41 ± 12.34	0.1991		
BMI (kg/m^2^)	25.68 ± 6.83	29.15 ± 5.96	0.0075		
MELD Score	21.94 ± 8.72	19.22 ± 8.12	0.1445		
Leukocytes (per nl)	7.46 ± 4.57	6.62 ± 5.01	0.2691		
Hemoglobin (g/dl)	9.11 ± 1.88	9.30 ± 2.30	0.840		
Thrombocytes (per nl)	121.42 ± 104.65	100.66 ± 58.50	0.9393		
Albumin (g/dl)	2.78 ± 0.62	3.07 ± 0.50	0.84		
Bilirubin (mg/dl)	8.19 ± 8.30	4.66 ± 6.57	0.24		
GOT (U/L)	95.18 ± 97.16	80.15 ± 111.79	0.3752		
GPT (U/L)	56.27 ± 56.92	51.97 ± 76.61	0.5339		
INR	1.70 ± 0.65	1.51 ± 0.46	0.2557		
Potassium (mmol/L)	3.87 ± 0.50	3.98 ± 0.59	0.4075		
Sodium (mmol/L)	136.12 ± 4.94	137.88 ± 5.40	0.1629		
Creatinine (mg/dl)	1.43 ± 0.88	1.37 ± 0.88	0.6439		
CRP (mg/dl)	4.66 ± 4.08	2.69 ± 2.62	0.0090		
Procalcitonin (ng/ml)	1.08 ± 2.04	0.37 ± 0.60	0.0050		
IL-17 (pg/ml)	331.56 ± 555.89	119.76 ± 376.12	0.0042		
IL-17E (pg/ml)	93.36 ± 226.34	94.66 ± 220.52	0.6739		
IL-17F (pg/ml)	102.81 ± 206.04	52.02 ± 157.36	0.3597		
IL-1RA (pg/ml)	1047.14 ± 994.05	453.80 ± 649.03	0.0209		
IL-1β (pg/ml)	60.20 ± 84.70	16.00 ± 45.40	0.0026		
IL-23 (pg/ml)	1797.25 ± 2788.65	551.07 ± 1632.37	0.0132		
IL-6 (pg/ml)	51.93 ± 56.03	24.84 ± 20.62	0.2619		
Ratio IL-17F/IL-17	38.21 ± 143.65	31.00 ± 90.97	0.1442		
Ratio IL-1RA/IL-1β	282.38 ± 578.54	287.21 ± 402.61	0.2713		

Variable	Infection n = 33	Infection (%)	No Infection n = 41	No Infection (%)	P-value
Presence of TIPS	4	12.12%	7	17.07%	0.789
Diabetes	9	27.27%	15	36.59%	0.547
Ascites at admission	27	81.82%	40	97.56%	0.057
Acute kidney failure					
No	16	48.48%	22	53.66%	0.904
Stage 1	6	18.18%	8	19.51%	
Stage 2	4	12.12%	5	12.20%	
Stage 3	7	21.21%	6	14.63%	
Hepatorenal syndrome	9	27.27%	10	24.39%	0.988
Hepatic encephalopathy at admission	11	33.33%	14	34.15%	1
ACLF	16	48.48%	16	39.02%	0.561
ACLF grade					
0	17	51.52%	25	60.98%	0.125
1	6	18.18%	12	29.27%	
2	9	27.27%	3	7.32%	
3	1	3.03%	1	2.44%	
Child–Pugh stage					
A	4	12.12%	3	7.32%	0.640
B	13	39.39%	20	48.78%	
C	16	48.48%	18	43.90%	

Data are expressed as mean ± SD.

*GGT* gamma-glutamyltransferase, *ALT* alanine aminotransferase, *AP* alkaline phosphatase, *AST* aspartate aminotransferase, *CRP* C-reactive protein, *G* grade, *INR* international normalised ratio, *MELD* model for end-stage liver disease, *TIPS* transjugular intrahepatic portosystemic shunt, *IL* interleukin, *ACLF* acute-on-chronic liver failure.

### Predictor screening process

The screening of predictors was performed in three sequential steps. Initially, independent predictors associated with the presence of infection were identified using univariate logistic regression analysis. The results of the unadjusted logistic regression, shown in Table 2, revealed a statistically significant association between infection and several biomarkers. Specifically, IL-23 (OR: 1.0002, 95% CI 1.0004–1.005, p = 0.029), IL-1RA (OR: 1.008, 95% CI 1.002–1.015, p = 0.005), IL-1β (OR: 1.01, 95% CI 1.003–1.021, p = 0.013), and CRP (OR: 1.20, 95% CI 1.03–1.43, p = 0.022) were all significantly associated with the presence of infection, whereas procalcitonin was not (p = 0.115).

**Table 2:** Unadjusted odds ratios (OR) and 95% confidence intervals (CI) from logistic regression models.

Variable	Odds ratio	Lower CI	Upper CI	P value
IL-17	1.001	1.00	1.002	0.086
IL-23	1.0002	1.0004	1.005	0.029
IL-1RA	1.008	1.002	1.015	0.005
IL-1β	1.01	1.003	1.021	0.013
Procalcitonin	1.69	1.05	4.26	0.115
Ferritin	1.001	0.99	1.02	0.106
CRP	1.20	1.03	1.43	0.022

*CRP* C-reactive protein, *IL* interleukin.

Subsequently, the biomarkers that demonstrated statistical significance in the univariate analysis were included in a multivariable logistic regression model, adjusting for pre-defined covariates: MELD score, age, presence of ACLF, and sex. After adjustment, only IL-1RA (p = 0.007) and IL-1β (p = 0.030) remained statistically significant, indicating their potential role as independent predictors of infection in patients with cirrhosis and acute decompensation (Table 3).

**Table 3:** Adjusted OR and 95% CI of the logistic regression models.

Model	Variable	Odds Ratio	Lower CI	Upper CI	P Value
Model 1	IL17	1.001	1.00002	1.002	0.086
	Age	0.969	0.928	1.009	0.138
	MELD-Score	1.040	0.956	1.132	0.350
	ACLF	0.937	0.224	3.947	0.928
	Sex	0.902	0.315	2.503	0.844
Model 2	IL1RA	1.0008	1.0002	1.001	0.007
	Age	0.968	0.927	1.009	0.140
	MELD-Score	1.026	0.941	1.120	0.545
	ACLF	1.0005	0.230	4.355	0.999
	Sex	1.011	0.347	2.892	0.983
Model 3	IL23	1.0002	0.999	1.0004	0.072
	Age	0.978	0.937	1.018	0.288
	MELD	1.028	0.944	1.119	0.510
	ACLF	0.975	0.237	4.064	0.972
	Sex	0.9931	0.3518	2.753	0.989
Model 4	IL1b	1.009	1.001	1.020	0.030
	Age	0.9783	0.936	1.020	0.306
	MELD-Score	1.026	0.943	1.117	0.536
	ACLF	0.967	0.232	4.044	0.963
	Sex	0.877	0.299	2.477	0.807
Model 5	MELD-Score	1.052	0.991	1.121	0.099
	Sex	1.490	0.510	4.423	0.464
	Age	0.964	0.922	1.005	0.096
	CRP	1.272	1.0002	1.556	0.087
Model 6	MELD-Score	1.028	0.967	1.094	0.367
	Sex	1.238	0.439	3.453	0.682
	Age	0.971	0.931	1.011	0.166
	Procalcitonin	1.657	1.031	4.269	0.120

*ACLF* acute-on-chronic liver failure, *CRP* C-reactive protein, *IL* interleukin.

Following this, and to improve the clinical utility, a binary classification approach (≤ cutoff vs. > cutoff) was applied, as previously described, to identify optimal thresholds for these biomarkers. The thresholds identified are presented in Supp. Table 3. The cut-off for IL-1RA was set at 1400 pg/mL, the cut-off for IL-1β was set at 3.7 pg/mL, and the cut-off for both CRP and PCT was set at 0.5 ng/ml (Supp. Figure 2). After dichotomization, we repeated both the univariate and multivariate analyses using logistic regression, with the results summarized in Supp. Table 4 and Table 4, respectively. Finally, six predictive models were developed, incorporating CRP, PCT (included despite their lack of individual statistical significance due to their widespread use in clinical practice), IL-1RA, IL-1β, and the previously mentioned clinical parameters.

**Table 4:** Multivariable adjusted odds ratios (OR) and 95% confidence intervals (CI) from logistic regression models for dichotomized variables.

Model	Variable	Odds ratio	Lower CI	Upper CI	P value
Model 1: IL1b–CRP	IL1b	7.759	2.039	39.349	0.005
	CRP	6.114	0.941	63.033	0.083
	MELD	1.007	0.916	1.104	0.866
	Sex	0.992	0.331	2.909	0.989
	Age	0.973	0.926	1.019	0.251
	ACLF	1.033	0.225	4.816	0.965
Model 2: IL1b–Procalcitonin	IL1b	5.481	1.506	24.068	0.014
	Procalcitonin	3.487	0.909	14.579	0.073
	MELD	0.969	0.875	1.071	0.549
	Sex	1.255	0.410	3.843	0.687
	Age	0.972	0.928	1.017	0.231
	ACLF	1.496	0.317	7.341	0.610
Model 3: IL1RA–Procalcitonin	IL1RA	7.154	1.800	37.052	0.008
	Procalcitonin	2.273	0.536	9.957	0.263
	MELD	0.980	0.886	1.081	0.701
	Sex	1.239	0.403	3.803	0.704
	Age	0.959	0.915	1.001	0.066
	ACLF	1.820	0.389	8.898	0.446
Model 4: IL1RA–CRP	IL1RA	10.676	2.685	58.585	0.001
	CRP	5.4110	0.813	58.078	0.111
	MELD	1.0012	0.912	1.095	0.977
	Sex	1.0997	0.361	3.316	0.865
	Age	0.9563	0.911	0.999	0.054
	ACLF	1.4937	0.323	7.275	0.607
Model 5: IL1RA	IL1RA	9.056	2.400	45.481	0.002
	MELD	1.004	0.916	1.097	0.929
	Sex	1.061	0.357	3.104	0.913
	Age	0.963	0.921	1.004	0.089
	ACLF	1.489	0.331	7.020	0.604
Model 6: IL1b	IL1b	5.842	1.686	24.341	0.008
	MELD	1.011	0.922	1.106	0.797
	Sex	0.974	0.333	2.785	0.961
	Age	0.980	0.938	1.023	0.374
	ACLF	1.036	0.235	4.632	0.962
Model 7: CRP	CRP_binary	3.310	0.650	25.607	0.1823
	MELD	1.039	0.958	1.131	0.349
	Sex	0.986	0.355	2.696	0.9784
	Age	0.967	0.925	1.008	0.124
	ACLF	1.015	0.250	4.124	0.982
Model 8: Procalcitonin	Procalcitonin_binary	3.860	1.105	14.788	0.031
	MELD	0.993	0.903	1.091	0.891
	Sex	1.283	0.448	3.694	0.639
	Age	0.867	0.925	1.008	0.124
	ACLF	1.025	0.250	4.124	0.982
Model 9: CRP + Procalcitiotin	Procalcitonin_binary	1.117	0.690	2.659	0.701
	MELD	1.047	0.982	1.120	0.161
	Sex	1.512	0.518	4.476	0.448
	Age	0.965	0.923	1.006	0.103
	ACLF	1.342	0.394	3.456	0.857
	CRP_binary	1.245	1.017	1.556	0.038

*ACLF* acute-on-chronic liver failure, *CRP* C-reactive protein, *IL* interleukin.

### Performance of the models

**Fig. 2:**
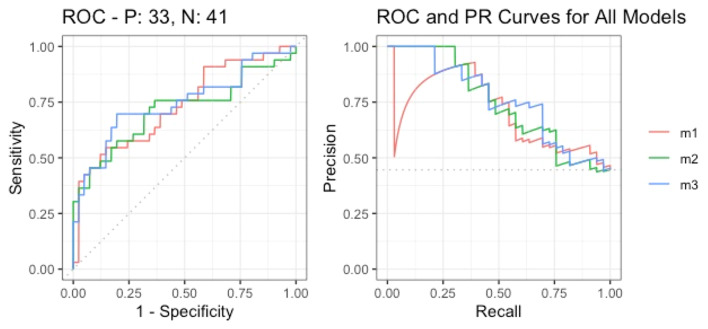
Receiver operating characteristic (ROC) curves for the three models with the highest AUC values:Red: IL-1RA + Procalcitonin, Green: IL-1RA + CRP, Blue: IL-1RA alone.

Model performance was assessed using AUC with 95% CI and key classification metrics, including accuracy, sensitivity, specificity, precision, negative predictive value, and F1-score (Table 5). The three models with the highest AUC and best discriminative ability included IL-1RA in combination with either CRP (AUC: 0.751, 95% CI 0.635–0.866) or PCT (AUC: 0.744, 95% CI 0.625–0.863), as well as IL-1RA alone (AUC: 0.762, 95% CI 0.652–0.872). These results suggest that IL-1RA-based models have superior performance in discriminating between infected and non-infected patients with cirrhosis. The corresponding ROC curves and diagnostic performance measures for all models are shown in Supp. Figures 3. and 4. Additionally, the AUC curves of the three best performing models are presented in Fig. 2.

**Table 5:** Diagnostic performance metrics of the models.

Model	AUC (95% CI)	Accuracy	Sensitivity	Specificity	Precision	NPV	F1 Score
Model 1: IL-1β + CRP	0.732 (0.615–0.849)	0.703	0.455	0.902	0.789	0.673	0.577
Model 2: IL-1β + Procalcitonin	0.715 (0.589–0.841)	0.703	0.545	0.829	0.72	0.694	0.621
Model 3: IL-1RA + Procalcitonin	0.744 (0.625–0.863)	0.72	0.547	0.854	0.75	0.7	0.632
Model 4: IL-1RA + CRP	0.751 (0.635–0.866)	0.716	0.576	0.829	0.731	0.708	0.644
Model 5: IL-1RA	0.762 (0.652–0.872)	0.74	0.548	0.854	0.75	0.7	0.632
Model 6: IL-1β	0.729 (0.612–0.847)	0.689	0.424	0.902	0.778	0.661	0.549
Model 7: CRP	0.670 (0.543–0.796)	0.649	0.485	0.78	0.64	0.653	0.552
Model 8: Procalcitonin	0.688 (0.565–0.811)	0.676	0.534	0.78	0.667	0.681	0.6
Model 9: CRP + Procalcitonin	0.693 (0.572–0.815)	0.649	0.545	0.732	0.621	0.667	0.581

*CRP* C-reactive protein; IL, interleukin.

**Fig. 3:**
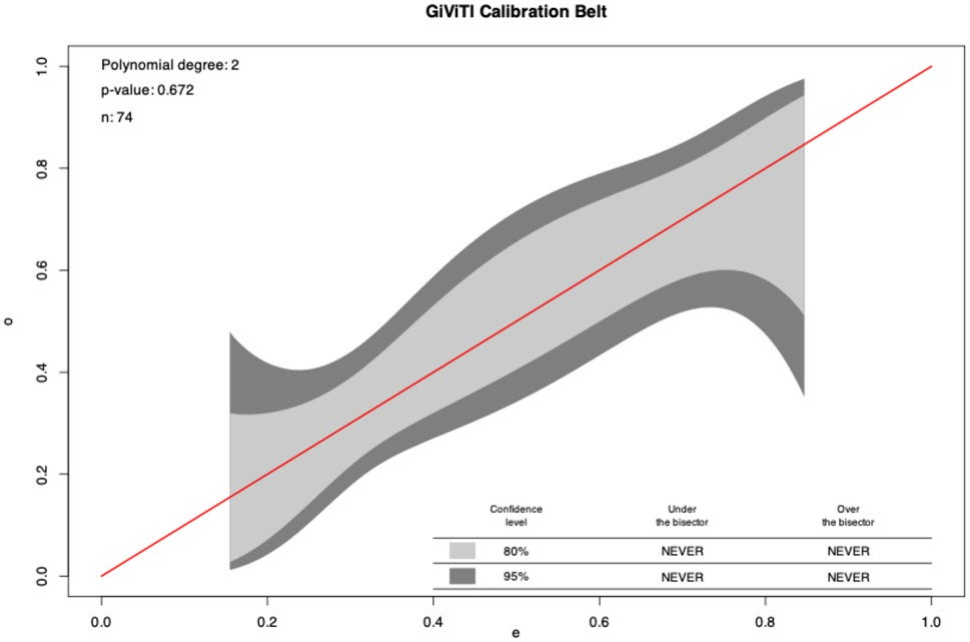
Calibration belt of the IL1RA-model.

**Fig. 4:**
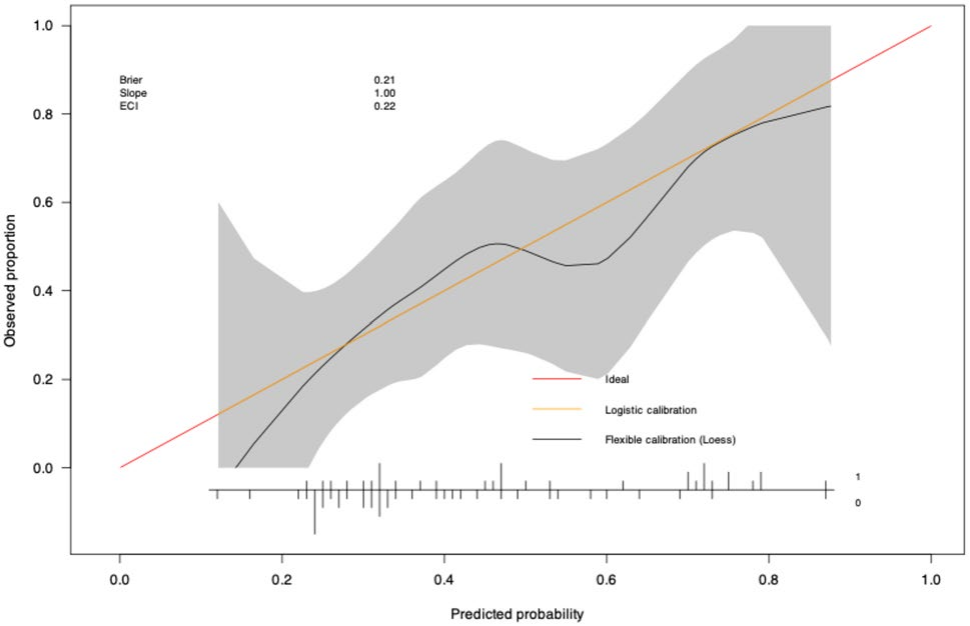
Calibration plot of the IL1RA-model.

Due to the superior performance of the IL1RA model, we decided to evaluate this model further. Calibration belt and calibration plot analyses showed a good calibration of the model (Figs. 3 and 4). The metrics are shown in Suppl. Table 5. The calibration belt analysis yields a p-value of 0.672, indicating good calibration. This interpretation is further supported by the corresponding plot, as shown in the figure. Notably, both the 80% and 95% calibration belts cover the bisector across all predicted probability values. This finding suggests that the model’s predictions align well with the observed results, demonstrating adequate internal calibration. The estimated slope coefficient in the calibration plot was 1, indicating that the model is neither overfitting (slope < 1) nor underfitting (slope > 1). This suggests that the model’s predictions closely align with the observed results.

To assess the robustness and generalizability of these models, internal validation was performed using bootstrap resampling. Specifically, 1,000 bootstrap iterations were performed, with each resample consisting of 74 randomly selected patients. This approach provided an empirical estimate of the model’s stability and variability across different subsets of the data. The results of the internal validation are presented in Suppl. Table 6. Mean AUC, sensitivity, specificity, and confidence intervals are shown in Suppl. Table 7. The final model is shown in Table 6. The adjusted calibration plot is shown in Suppl. Figure 5. DeLong’s test showed no statistically significant AUC differences between the final IL-1RA model and CRP alone or procalcitonin alone (*p* = 0.3694 and 0.5067, respectively) (Suppl. Table 7).

**Table 6:** Multivariable adjusted (after bootstrapping) odds ratios (OR) and 95% confidence intervals (CI) from logistic regression.

Variable	Coefficient	SE	Lower CI	Upper CI	Odds Ratio	Lower CI	Upper CI	P Value
IL1RA_binary	0.6985	0.2262	0.3300	1.1296	2.0107	1.3910	3.0944	0.02
MELD	0.0146	0.0116	− 0.0105	0.0334	1.0147	0.9896	1.0340	0.20
Sex	0.0498	0.2325	− 0.4198	0.4886	1.0510	0.6572	1.6301	0.90
Age	− 0.0126	0.0082	− 0.0294	0.0019	0.9875	0.9710	1.0019	0.14
ACLF	0.0872	0.2110	− 0.2972	0.5026	1.0911	0.7429	1.6530	0.62

*ACLF* acute-on-chronic liver failure, *CRP* C-reactive protein, *IL* interleukin.

### Risk stratification and clinical application

**Fig. 5:**
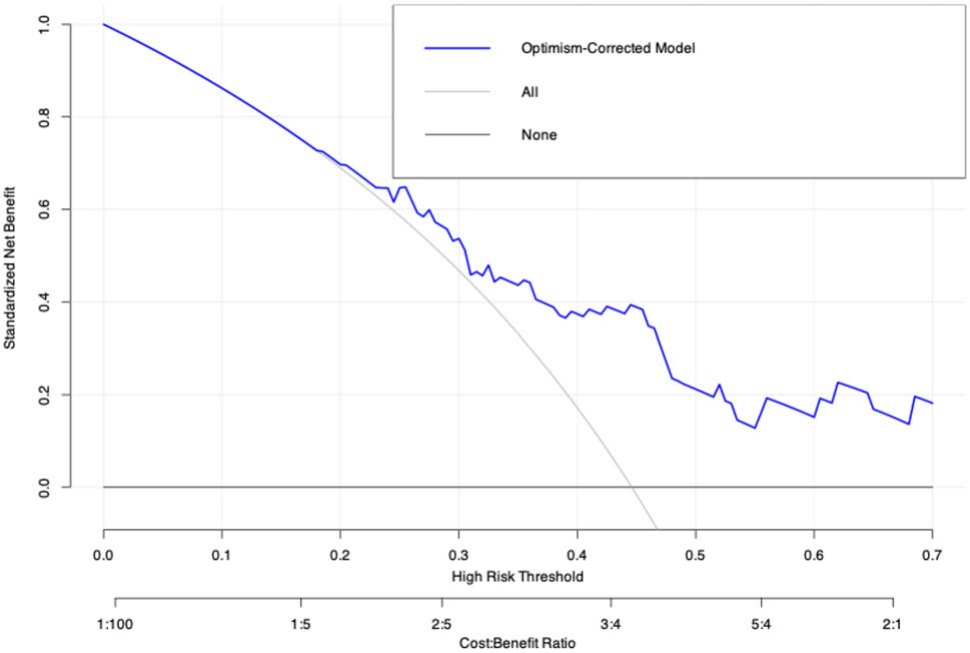
RCA of the IL1RA-model after bootstrapping. The figure presents the net benefit analysis using a decision curve. This analysis clearly demonstrates the superiority of our model compared to the strategy of treating all patients with antibiotics. It highlights the model’s ability to balance the benefits and harms of antibiotic therapy across different probability thresholds. For example, at a threshold probability of 0.45, the net benefit difference between our model and the “treat all” approach is 0.39. This means that using our model instead of treating all patients increases the number of correctly treated bacterial infections (true positives) by 39 per 100 patients, without increasing the number of unnecessary antibiotic treatments (false positives).

Furthermore, we conducted a net benefit analysis using a decision curve (Fig. 5). This analysis demonstrates the superiority of our model compared to the strategy of treating all patients with antibiotics. For example, at a threshold probability of 0.45, the net benefit difference between our model and the “treat all” approach is 0.39. This means that using our model instead of treating all patients increases the number of correctly treated bacterial infections (true positives) by 39 per 100 patients, without increasing the number of unnecessary antibiotic treatments (false positives).

To improve the clinical applicability of the model, cut-off thresholds were established to achieve 80% sensitivity and 80% specificity. These thresholds were selected to balance detection of infection (sensitivity) with minimization of false-positives results (specificity). Using these cut-offs and coefficients derived from bootstrap regression, we developed a practical risk stratification model for clinical use. The model is readily accessible via an application, available at the following URL: [https://gdkonsta.shinyapps.io/InfectionPredictionApp/].

## Discussion

Bacterial infections are the most common trigger of acute decompensation in cirrhosis^10–12^. The CANONIC study identified bacterial infections as a contributing factor in 33% of ACLF cases^8,28^. In recent years, it has become increasingly evident that the immune environment in cirrhosis is not uniform. Instead, it represents a delicate equilibrium between pro-inflammatory and anti-inflammatory states^14^. This condition, known as CAID^15^, plays a key role in the increased susceptibility of patients with cirrhosis to bacterial infections. CAID encompasses a spectrum of dynamic and reversible immune alterations, including low-grade systemic inflammation, which characterizes patients with compensated cirrhosis, and high-grade systemic inflammation, which serves as a key pathogenic driver of organ failure^14,15,29^.

In the latter state, continuous exposure to pathogen associated molecular patterns (PAMPs) causes a gradual shift in the immune response. Impaired bacterial opsonization and immune cell paralysis further exacerbate immune suppression, significantly increasing susceptibility to infection^14^. Early diagnosis and timely treatment of infections are essential in the management of patients with AD, as infections in the setting of an impaired immune system can significantly deteriorate outcomes and increase mortality^30^. Current guidelines recommend that every patient admitted with AD or who develops AD during hospitalization should undergo a comprehensive diagnostic assessment to identify potential triggers, with a particular focus on bacterial infections ^18,21^. In this setting, empirical broad-spectrum antibiotics are initiated immediately upon suspicion of infection, adapted to local resistance patterns. However, conventional microbiological techniques can take up to five days to identify pathogens and determine their susceptibility, leading to prolonged exposure to antibiotics, which in turn may accelerate antimicrobial resistance and increase susceptibility to infection^31,32^. This is further underlined by our cohort results, where 19 patients who were initially free of infection but received antibiotics on admission developed infection-triggered AD within three months.

Another challenge in diagnosing infection in patients with cirrhosis is the limited reliability of conventional markers of inflammation. Leukocyte count, a standard marker of infection in the general population, is often reduced in cirrhosis due to hypersplenism^33^ or may be elevated due to acute steatohepatitis^34^. Similarly, CRP is directly influenced by liver function, with its production declining as liver function deteriorates, thereby compromising its diagnostic and prognostic value in patients with advanced cirrhosis^35^. Meanwhile, PCT also has an unclear prognostic role in cirrhosis. The EASL guidelines propose CRP ≥ 10 mg/L and PCT ≥ 0.49 ng/mL as cut-off values for the assessment of infection, but these recommendations are mainly based on older studies^18,35,36^. The identification of reliable biomarkers that can effectively stratify patients with cirrhosis based on their risk of infection at an early stage remains a critical unmet need^18,37^.

We aimed to address this need by evaluating various circulating interleukins in the serum of patients with liver decompensation to gain insight into their immune profile, with a particular focus on the IL-17 and IL-1 cytokine families. Additionally, we explored their potential as biomarkers for the early diagnosis of bacterial infections. We chose to examine these specific cytokines due to the critical role of the IL-1 network in bacterial clearance and recruitment of immune cells^38,39^, whereas Th17-derived cytokines, such as IL-17, support mucosal immunity by promoting neutrophil recruitment and stimulating the production of antimicrobial peptides^40,41^. While this interleukin axis has previously been studied in non-infectious conditions in the context of liver cirrhosis, its role in infection-related decompensation has not yet been characterized^42^.

Specifically, we investigated whether IL-1RA, previously described in other infectious contexts, provides added diagnostic value in this unique clinical setting^43,44^.Through rigorous statistical analyses, we established a classification based on serum IL-1RA levels, demonstrating IL-1RA as a reliable biomarker for the diagnosis of bacterial infection. This finding aligns with the known anti-inflammatory role of IL-1RA^43,44^. Its role as a biomarker has been investigated in the context of community-acquired bacterial infections^44^. Both IL-1 and IL-1RA are primarily produced in human monocytes, macrophages, and neutrophils during various infections, with the liver contributing to IL-1RA production in a delayed manner following LPS exposure^14^. As a natural antagonist of IL-1, IL-1RA promotes the expression of anti-inflammatory cytokines such as IL-10 and IL-4, while simultaneously inhibiting the production of pro-inflammatory cytokines such as IL-1, and IL-6^45^. By competitively binding to IL-1 receptors without triggering downstream signaling, IL-1RA plays a critical role in modulating inflammation^46,47^. Notably, our model demonstrated superior prognostic performance compared to CRP and PCT, with an AUROC of 0.76 compared to 0.66 and 0.68, respectively. Furthermore, the robustness of the prognostic accuracy and calibration of our model was validated by bootstrap analysis, effectively correcting for potential optimism in performance estimates. Our model has the potential to accurately detect patients with bacterial infection-associated decompensation, providing crucial clinical insight into the management and treatment of these individuals.

This study has certain limitations that should be acknowledged. First, as a single-center study, our findings require external validation before our model can be integrated into clinical practice. Given the exploratory nature of our research and the use of cytokine measurements that are not routinely assessed in clinical settings, we were unable to include an external validation cohort with a comparable sample size and patient characteristics. Despite the fact that our study was conducted without a prespecified power calculation, a post-hoc power analysis considering an expected prevalence of 30% from the literature and a prevalence of 44% prevalence in our cohort, yielded a power of 73.1%. Although ideal predictive modeling involves both internal and external validation, we sought to address this limitation through bootstrap analysis, which is widely regarded as the statistical gold standard when an independent validation cohort is not available. All patients in this study received empiric antibiotic therapy before biomarker sampling, in line with current clinical practice guidelines and because initial suspicion of infection was supported by elevated CRP and/or leukocyte counts. Therefore, antibiotic-related modulation of cytokine levels, including IL-1RA, cannot be excluded. Prospective studies measuring IL-1RA before and after antibiotic initiation are warranted to clarify its diagnostic behavior in the absence of antimicrobial influence.

Furthermore, to increase the internal validity of our findings, we applied strict exclusion criteria, resulting in a homogeneous study population. However, this approach inevitably limits the generalizability of the model, emphasizing the need for further validation in more diverse patient populations to confirm its broader clinical applicability. Lastly, although the sample size was relatively small, it is important to highlight that this was a prospective study that involved complex cytokine measurements, an area where there is currently limited pre-existing data with a comparable research focus. Although pairwise DeLong tests did not yield statistically significant differences in AUC, the consistent numerical superiority of the IL-1RA model together with a favorable net benefit on decision curve analysis supports the potential clinical utility of IL-1RA for early infection detection in AD/ACLF.

## Conclusion

In conclusion, accurate detection of bacterial infections in patients with decompensated liver disease is crucial to reduce mortality rates. Our newly developed model, designed for a broad spectrum of patients with AD and ACLF, demonstrates superior predictive performance compared to conventional biomarkers such as CRP and PCT. Although IL-1RA itself is not a novel biomarker for infections, its application and validation within this complex immunological context represent an important step toward personalized patient management, enabling earlier infection detection, optimized antibiotic use, and ultimately improved clinical outcomes that may reduce the overall disease burden.

Looking ahead, we plan to conduct multi-center validation studies across diverse patient populations and real-world clinical settings to further establish the robustness and clinical applicability of our model. By leveraging these advanced methods, we seek to deepen our understanding of immune dysregulation in cirrhosis, refine biomarker driven diagnostic and therapeutic strategies, and ultimately contribute to more precise and effective patient care.

## Supplementary Information

Supplementary Information.

## Data Availability

The datasets used and analysed during the current study available from the corresponding author on reasonable request.
