# Supplementary material for: IL-1 receptor antagonist as a diagnostic biomarker for bacterial infections in acute decompensation of cirrhosis
Source: Sci Rep. 2025 Dec 7;15:43266. doi: 10.1038/s41598-025-30647-5 (PMC12685954; doi:10.1038/s41598-025-30647-5)
Supplement: Supplementary file 1 — Supplementary Information. [file 41598_2025_30647_MOESM1_ESM.docx]

**Supplementary Figure 1:** Distribution of various interleukins by infection status. The Wilcoxon rank test was used to evaluate differences between groups. Patients without infection are represented as "0" (blue), while patients with infection are labeled as "1" (yellow). The interleukin concentrations are expressed in pg/mL.


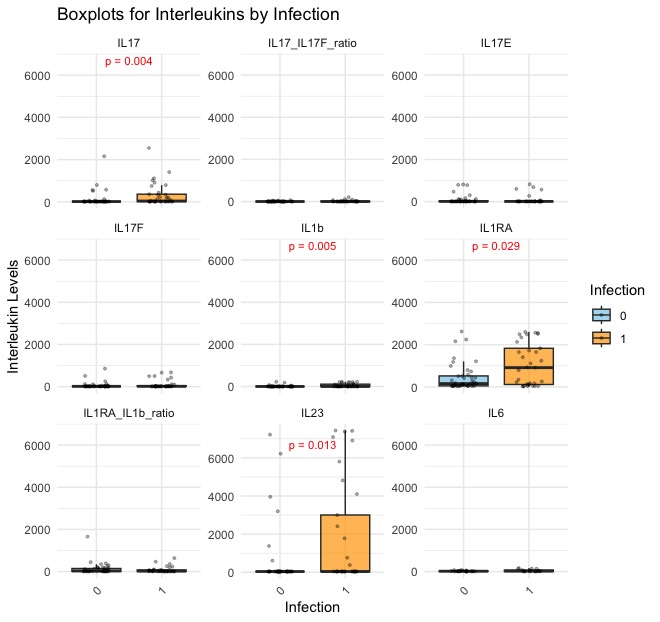


**Supplementary Figure 2:** AIC values at varying of IL1RA, IL1b, CRP, PCT concentrations

**Supplementary Figure 3**: Receiver Operating Characteristic (ROC) Curves for All Models (1-9)


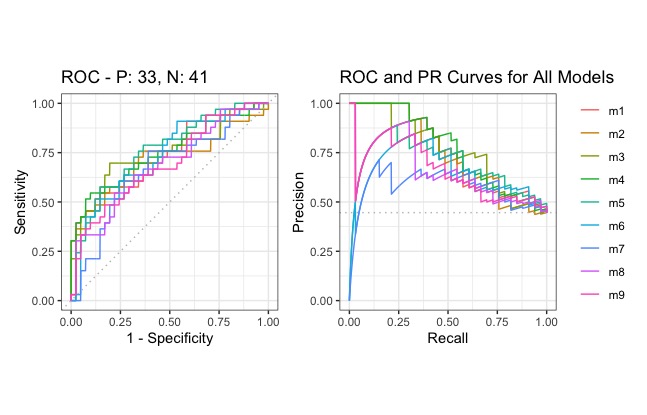


**Supplementary Figure 4**: Diagnostic Performance Measures for All Models The ranking follows the model numbering, which corresponds to the order presented in Tables 4 and 5. This figure provides a comprehensive overview of the diagnostic performance metric


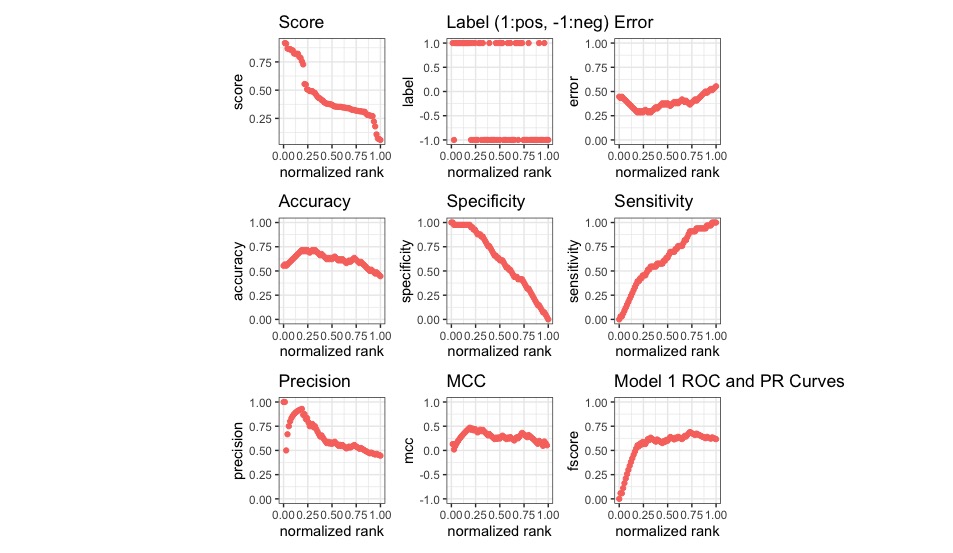

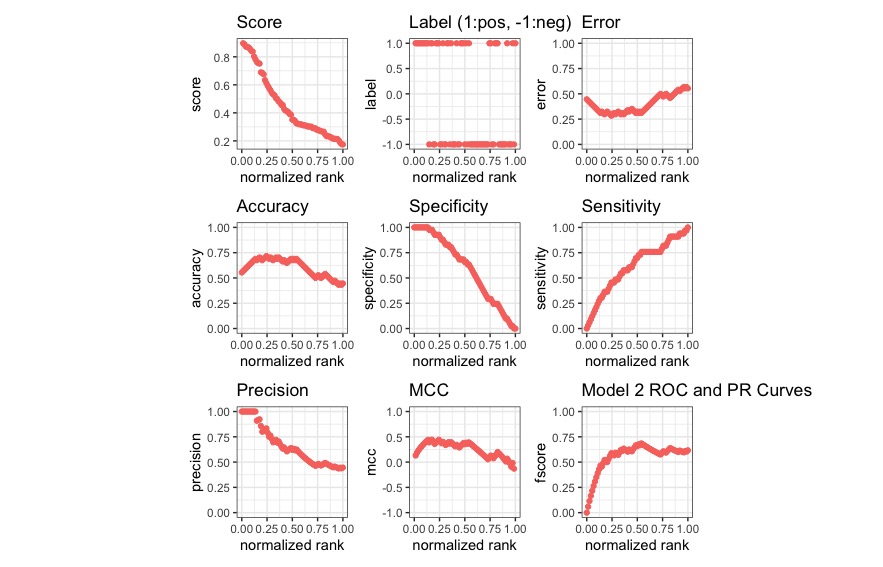

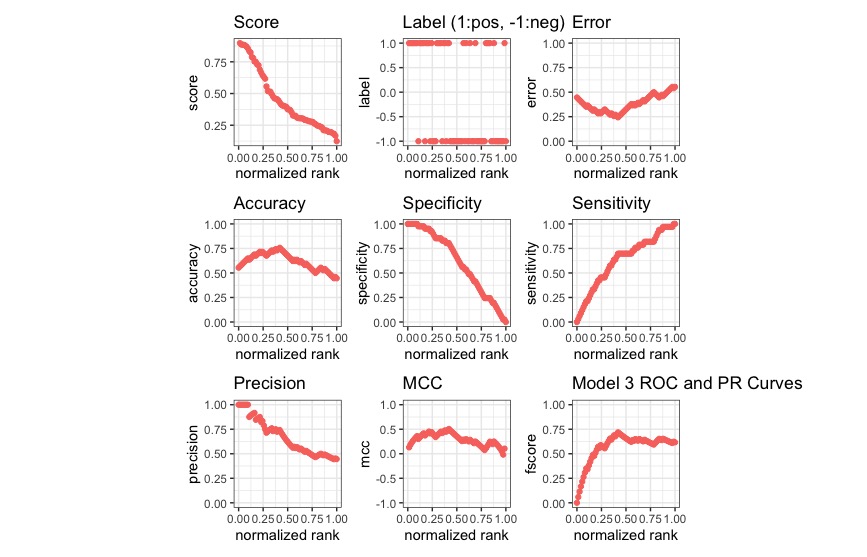

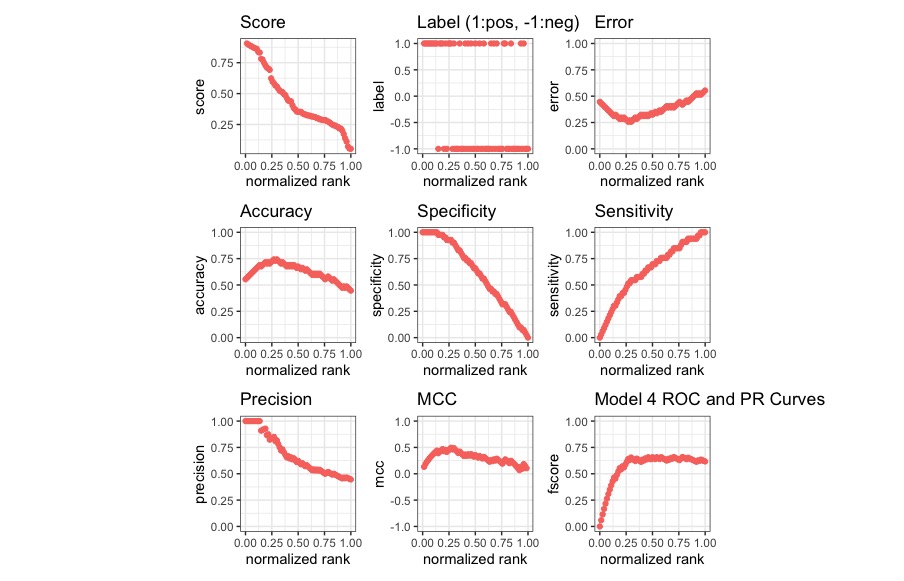

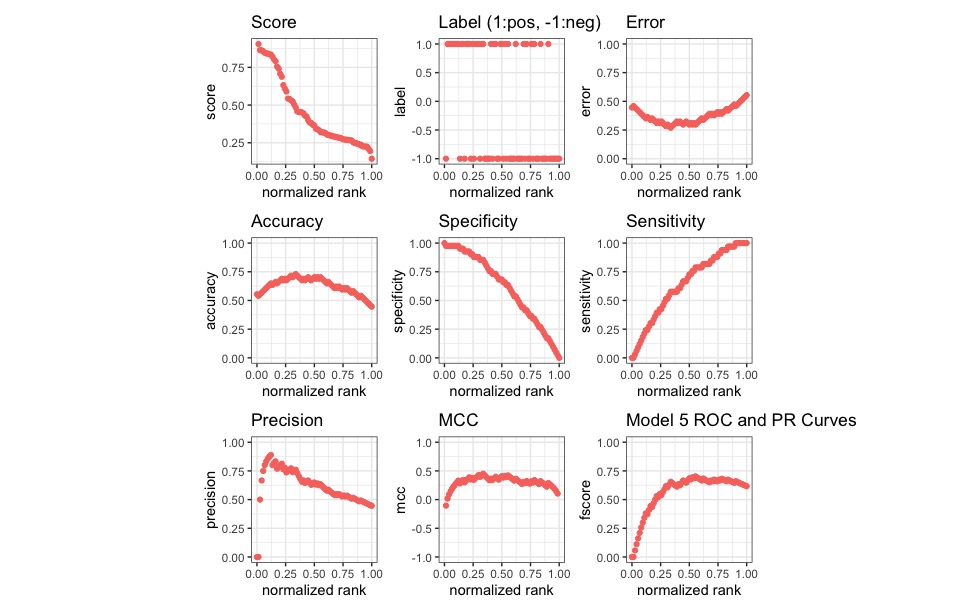

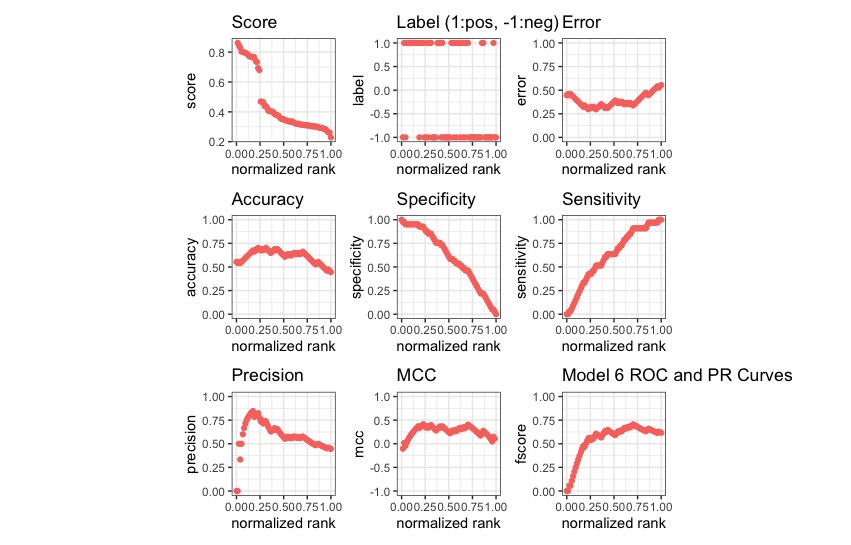

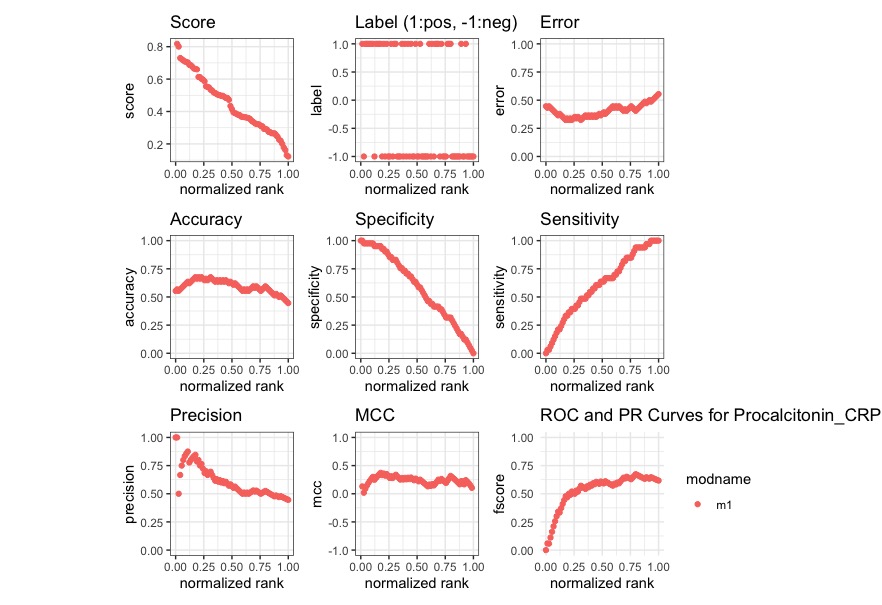

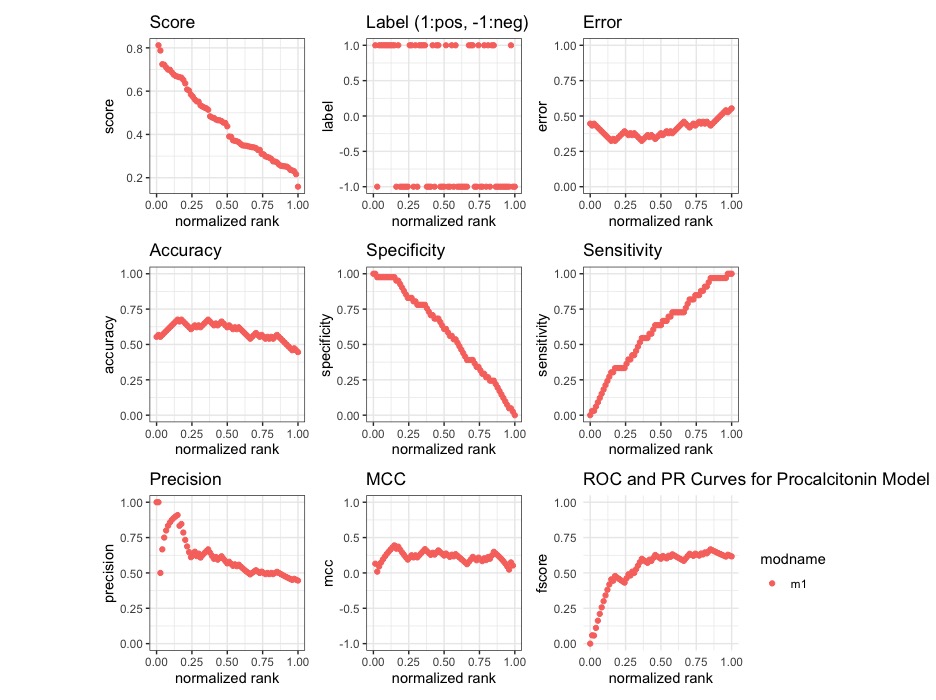

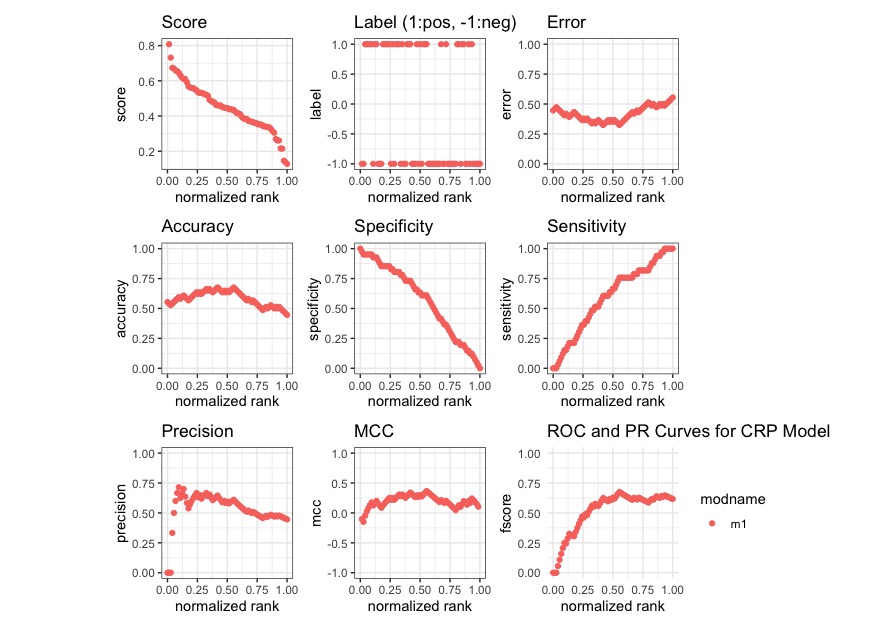


**Supplementary Figure 5: Calibration Plot of the IL1RA-Model after bootstrapping.**

**Supplementary Table 1.** Cause of Cirrhosis

| Liver Cirrhosis Cause | No Infection | Infection | Total | P-value |
| --- | --- | --- | --- | --- |
| AIH | 1 | 1 | 2 | > 0.05 |
| Alcohol | 17 | 18 | 35 | > 0.05 |
| Autoimmune Hepatitis | 1 | 0 | 1 | > 0.05 |
| Biliary atresia | 0 | 1 | 1 | > 0.05 |
| Budd-Chiari Syndrome | 1 | 0 | 1 | > 0.05 |
| Cryptogenic | 3 | 2 | 5 | > 0.05 |
| Hemochromatosis | 0 | 1 | 1 | > 0.05 |
| Hep B/D coinfection | 3 | 0 | 3 | > 0.05 |
| Hep C | 1 | 0 | 1 | > 0.05 |
| MASH | 9 | 2 | 11 | 0.01 |
| PBC | 1 | 1 | 2 | > 0.05 |
| PSC | 3 | 6 | 9 | > 0.05 |
| Secondary sclerosing cholangitis | 1 | 1 | 2 | > 0.05 |
|  |  |  |  |  |
| *AIH, autoimmune hepatitis; MASH, Metabolic dysfunction-associated steatohepatitis; PBC, primary biliary cholangitis; PSC, primary sclerosing cholangitis* | | | | |
| Supplementary Table 2. Site of Infection | | |  |  |
| Infection Type | **Count** | **Percentage** |  |  |
| Unclear focus | 8 | 24 |  |  |
| Urinary tract infection | 7 | 21 |  |  |
| Cholangitis | 5 | 11 |  |  |
| Pneumonia | 8 | 24 |  |  |
| Spontane bacterial peritonitis | 2 | 6 |  |  |
| Clostridial enteritis | 2 | 6 |  |  |

| Organism isolated | Count | Percentage |
| --- | --- | --- |
| Escherichia coli | 10 | 37 |
| Enterococcus faecium | 5 | 19 |
| Staphylococcus aureus | 3 | 12 |
| Klebsiella oxytoca | 2 | 8 |
| Clostridioides difficile | 2 | 8 |
| Pseudomonas aeruginosa | 1 | 4 |
| Acinetobacter baumannii | 1 | 4 |
| Staphylococcus haemolyticus | 1 | 4 |
| Serratia marcescens | 1 | 4 |

**Supplementary Table 3. Optimal Biomarker Thresholds and AIC Values**

| Biomarker | Best Threshold | Minimum AIC |
| --- | --- | --- |
| IL-1β (pg/mL) | 3.8 | 94.812 |
| IL-1RA (pg/mL) | 1400.0 | 94.168 |
| CRP (mg/dL) | 0.5 | 12.179 |
| Procalcitonin (ng/ml) | 0.5 | 71.908 |

*ACLF, acute-on-chronic liver failure; CRP, C-reactive protein; IL, interleukin*

**Supplementary Table 4**. Unadjusted Odds Ratios (OR) and 95% Confidence Intervals (CI) from Logistic Regression Models for dichotomized variables

| Variable | Odds Ratio | Lower CI | Upper CI | P Value |
| --- | --- | --- | --- | --- |
| IL-1RA (binary) | 4.86 | 1.67 | 15.67 | 0.005 |
| CRP (binary) | 2.65 | 0.56 | 19.05 | 0.251 |
| Procalcitonin (binary) | 3.15 | 1.10 | 9.66 | 0.035 |
| IL-1β (binary) | 1.76 | 1.75 | 30.28 | 0.008 |

*ACLF, acute-on-chronic liver failure; CRP, C-reactive protein; IL, interleukin*

**Supplementary Table 5. Calibration Metrics for IL1RA model**

| Metric | Description | Value |
| --- | --- | --- |
| Dxy | Somers' D | 0.454 |
| R2 | Nagelkerke R² | 0.198 |
| D | D-Statistic | 0.146 |
| D:Chi-sq | D-Statistic Chi-Square | 11.862 |
| D:p | D-Statistic p-value | 0.0005 |
| U | U-Statistic | -0.027 |
| U:p | U-Statistic p-value | 0.999 |
| Q | Q-Statistic | 0.173 |
| Brier | Brier Score | 0.209 |
| Intercept | Calibration Intercept | 0.00 (-0.50 – 0.50) |
| Slope | Calibration Slope | 1.00 (0.38 – 1.62) |
| Emax | Maximum Absolute Calibration Error | 0.0006 |
| Brier scaled | Scaled Brier Score | 0.1540 |
| Eavg | Average Calibration Error | 0.033 |
| ECI | Expected Calibration Index | 0.221 |

**Supplementary Table 6. Calibration Metrics for IL1RA model after optimism correction**

| Metric | Description | Value |
| --- | --- | --- |
| Dxy | Somers' D | 0.310 |
| R² | Nagelkerke R² | 0.07 |
| Intercept | Calibration intercept | -0.070 |
| Slope | Calibration slope | 0.692 |
| Emax | Maximum absolute calibration error. | 94.0 |
| D | D-Statistic | 0.029 |
| U | U-Statistic | 0.056 |
| Q | Q-Statistic | -0.027 |
| B | Brier Score | 0.246 |
| g | g-Mean | 0.497 |
| gp | Scaled g-Mean | 0.154 |
| Emax | Emax | 0.836 |
| Eavg | Eavg | 0.426 |
| ECI | ECI | 0.231 |

**Supplementary Table 7. Diagnostic Performance Metrics of the IL1RA Model (Corrected For Optimism)**

|  | AUC | AUC Lower | AUC Upper | Accuracy | Sensitivity | Specificity | PPV | NPV | F1 Score |
| --- | --- | --- | --- | --- | --- | --- | --- | --- | --- |
| IL1RA Model Performance (Corrected For Optimism) | 0.729 | 0.613 | 0.846 | 0.635 | 0.424 | 0.805 | 0.636 | 0.635 | 0.509 |

**Supplementary Table 8. Comparison of the final IL-1RA model with conventional biomarkers using DeLong’s test**

| **Comparison** | **p-value** |
| --- | --- |
| Final model† vs **CRP (alone)** | 0.3694 |
| Final model† vs **Procalcitonin (alone)** | 0.5067 |
